# Are the SSB-Interacting Proteins RecO, RecG, PriA and the DnaB-Interacting Protein Rep Bound to Progressing Replication Forks in *Escherichia coli*?

**DOI:** 10.1371/journal.pone.0134892

**Published:** 2015-08-05

**Authors:** Esma Bentchikou, Carine Chagneau, Emilie Long, Mélody Matelot, Jean-François Allemand, Bénédicte Michel

**Affiliations:** 1 Institute for Integrative Biology of the Cell (I2BC), CEA, CNRS, Université Paris Sud, Gif sur Yvette, France; 2 Laboratoire de Physique Statistique, Département de Physique, UMR 8550 CNRS, Ecole Normale Supérieure, Universités Pierre et Marie Curie and Paris Diderot, Paris, France; 3 IBENS, Département de Biologie, UMR 8542 CNRS, Ecole Normale Supérieure, Paris, France; University of Iowa, UNITED STATES

## Abstract

In all organisms several enzymes that are needed upon replication impediment are targeted to replication forks by interaction with a replication protein. In most cases these proteins interact with the polymerase clamp or with single-stranded DNA binding proteins (SSB). In *Escherichia coli* an accessory replicative helicase was also shown to interact with the DnaB replicative helicase. Here we have used cytological observation of Venus fluorescent fusion proteins expressed from their endogenous loci in live *E*. *coli* cells to determine whether DNA repair and replication restart proteins that interact with a replication protein travel with replication forks. A custom-made microscope that detects active replisome molecules provided that they are present in at least three copies was used. Neither the recombination proteins RecO and RecG, nor the replication accessory helicase Rep are detected specifically in replicating cells in our assay, indicating that either they are not present at progressing replication forks or they are present in less than three copies. The Venus-PriA fusion protein formed foci even in the absence of replication forks, which prevented us from reaching a conclusion.

## Introduction

Replication is a universal process ensuring genome duplication. It involves the concomitant synthesis of a continuous leading-strand and a discontinuous lagging-strand, synthesized as Okazaki fragments that are joined by ligase. In *Escherichia coli*, replication is catalyzed by a complex called the replisome composed of (i) the polymerase III holoenzyme (Pol III HE) catalyzing DNA synthesis, (ii) the helicase DnaB that opens the double-stranded DNA template and (iii) a primase that synthesizes primers for replication initiation and for the synthesis of each Okazaki fragment [1]. Pol III HE contains three copies of the polymerase III subunit DnaE (also called α), two for coordinate leading- and lagging-strand synthesis and a spare one [2, 3]. Each active DnaE subunit is stabilized on DNA by a β-clamp (dimer of the DnaN protein), which encircles double-stranded DNA (dsDNA) after being loaded by a molecular complex called the clamp loader. The clamp loader is composed of a pentameric core complex (three DnaX subunits (τ), one HolA (δ) and one HolB (δ’)), and of the HolCD complex (χψ), present as one copy per replisome *in vitro* and four copies *in vivo* [1, 3]. In addition, the single-stranded DNA (ssDNA) transiently made on the lagging-strand template during Okazaki fragments synthesis is covered by SSB protein tetramers.

The exact number of SSB tetramers per replication fork has not been experimentally determined but it can be estimated. *In vitro* SSB binds ssDNA in two different modes depending on salt and concentration conditions, SSB_65_ and SSB_35_, which correspond to either 65 or 35 nucleotides wrapped around each SSB tetramer, respectively [4, 5]. Since Okazaki fragments are about 1 to 2 kb in length, the SSB_65_ binding mode implies a maximum of ~15–30 tetramers per fork (~60–120 SSB molecules), while the SSB_35_ binding mode implies the presence of ~28–56 tetramers (~112–224 SSB molecules). The maximum number of SSB molecules per fork can thus be estimated to range from 60 to 224. *In vitro*, SSB interacts through its C-terminal amino acidic tail with at least 15 proteins involved in DNA recombination, replication restart, or DNA repair [6]. Whether these proteins bind SSB constitutively at replication forks, traveling with progressing forks or whether they interact with SSB when needed is an open question. Here, we addressed this question by cytological observation of three SSB-interacting fluorescent molecules in live *E*. *coli* cells. PriA is essential for replication restart from blocked replication forks and recombination intermediates [7]. RecG is a multifunctional helicase involved in replication and recombination [8]. RecO is a pre-synaptic protein that promotes RecA binding to ssDNA gaps during recombinational gap repair [9]. We describe the construction of strains that express Venus-PriA, Venus-RecG or Venus-RecO fluorescent proteins from their endogenous chromosomal loci. These proteins were analyzed with a microscope that allows the detection of as few as three proteins.

SSB is not the only replisome component that interacts with replication or repair proteins. The DnaN clamp interacts with several classes of enzymes as for example bypass polymerases, mismatch repair proteins, sister-chromatids cohesion proteins and ligase [10, 11]. The DnaB helicase interacts with the accessory helicase Rep, which removes proteins and particularly RNA polymerases from the path of replication forks [12, 13]. We describe here the construction of a *Venus*-*rep* gene fusion expressed from the endogenous chromosomal locus and the use of microscopy to test for the presence of Rep at progressing replication forks. DnaB is a hexamer and thus a maximum of six Rep proteins can be expected if all DnaB molecules are Rep-bound.

Quantification of replisome components *in vivo* by microscopy showed the presence of one HolA, three DnaX proteins and four HolCD complexes in active *E*. *coli* replisomes [3, 14]. The microscope that we constructed allows us to detect replication-specific fluorescent foci in growing cells expressing DnaX-Venus, DnaX-YPet (three molecules) or HolD-YPet (four molecules), but not HolA-Venus (one molecule). Therefore, our technique should allow us to detect SSB- or DnaB-bound molecules if they travel with forks in 3 copies or more. We did not detect specific foci with the two SSB-interacting proteins Venus-RecO and Venus-RecG, and did not detect the DnaB interacting protein Venus-Rep, suggesting that either these proteins do not travel with forks or they do so in less than three copies. Our Venus-PriA fusion formed foci in replicating cells but also in the absence of replication, preventing us from reaching a conclusion for this protein.

## Materials and Methods

### Strains and plasmids

Strain carrying the *holD*-YPet fusion was obtained by P1 transduction of the fusion gene from the AB1157 background (a gift of R. Reyes-Lamothe and D. Sherratt, [15]) into a MG1655 background, and the *dnaX*-Ypet fusion was constructed as described [15]. In both strains the adjacent KanR marker was excised by FRT recombination [16], yielding JJC5349 and JJC5956, respectively. All other fusion genes were constructed in the laboratory. We will only describe in detail below the construction of strains used for the microscopy experiments reported here, but all plasmids and strains used for these strain constructions are also available on request.

#### holA-Venus

The 11 amino acids linker used by Reyes-Lamothe et al [15] linked to Venus and an adjacent Cm^R^ gene (originally from pKD3) were amplified from a strain carrying a functional *recD*-*venus* fusion, using the following oligonucleotides: 3’ ATC TCT TCT GTT GTG CCA TAA ACC CCT GGC GGA CGT ATT TAT CGA CGG T TCG GCT GGC TCC GCT GCT G 3’ and 5’ GTG CAC CGG ATC AAA GGT GCC GCC AAA CAG AGC CTG TAA AGA TTT CAT GCC ATG GTC CAT ATG AAT ATC CTC CTT 3’. The PCR product carries the last 50 nucleotides of *holA* linked by the 11 amino acids linker to Venus, the Cm^R^ gene from pKD3 and ends with 50 nucleotides localized after *holA*. Transformation by electroporation of DY330 [17] competent cells creates a strain in which the *holA*-Venus C-ter fusion is adjacent to a downstream Cm^R^ gene (JJC6039). Because the original construction lacked the stop codon after Venus, in a second step two stop codons were introduced together with a Kan^R^ gene, using the following oligonucleotides: 3’ TCG TGA CCG CCG CCG GGA TCA CTC TCG GCA TGG ACG AGC TGT ACA AGT AAT GAG CAA GGG CTG CTA AAG GAA G 5’ and 3’ GTG CAC CGG ATC AAA GGT GCC GCC AAA CAG AGC CTG TAA AGA TTT CAT AGA ACT CCA GCA TGA GAT CC 5’ to PCR amplify the Kan^R^ gene from pKD4 and transform DY330 *holA-Venus* (lacking stop codon) by electroporation with the PCR product. The fusion was verified by sequencing and P1 transduced, selecting Kan^R^, in MG1655. The transductant JJC6633 was verified by PCR and used for microscopy. As shown in S1A Fig, JJC6633 showed 100% plating efficiency on LB and on MM at 30°C, 37°C and 42°C, showing that the *holA-Venus* fusion is functional.

#### dnaX-Venus

Venus and the adjacent Cm^R^ gene were amplified by PCR from a strain carrying a functional *recD*-*venus* C-ter fusion using the following oligonucleotides: 5’ TG CGT CGG TTC TTC GAT GCG GAG CTG GAT GAA GAA AGT ATC CGC CCC ATT TCG GCT GGC TCC GCT GCT G 3’ and AAA CAT AGG TTT CTC TCT CAA TCA CGT TAA GGA TGA CGA ACG TAA GCT GT G CCA TGG TCC ATA TGA ATA TCC 3’. The PCR product carries the last 50 nucleotides of *dnaX* linked by the 11 amino acids linker to Venus, the Cm^R^ gene from pKD3 and ends with 50 nucleotides localized after *dnaX*. Transformation by electroporation of DY330 creates a strain in which the *dnaX*-Venus C-ter fusion is adjacent to a downstream CmR gene. Because the original construction lacked the stop codon after Venus, in a second step two stop codons were introduced together with a Kan^R^ gene, using the following oligonucleotides: 3’ TCG TGA CCG CCG CCG GGA TCA CTC TCG GCA TGG ACG AGC TGT ACA AGT AAT GAG CAA GGG CTG CTA AAG GAA G 5’ and 3’ AAA CAT AGG TTT CTC TCT CAA TCA CGT TAA GGA TGA CGA ACG TAA GCT GT A GAA CTC CAG CAT GAG ATC C 5’ to PCR amplify the Kan^R^ gene from pKD4 and transform DY330 *dnaX-Venus* (lacking stop codon) by electroporation with the PCR product. The fusion was verified by sequencing and P1 transduced, selecting Kan^R^, in MG1655. The transductant JJC6632 was verified by PCR and used for microscopy. As shown in S1A Fig, JJC6632 was fully viable on LB and on MM at 30°C, 37°C and 42°C.

pGBKD3-2 is a hybrid vector composed of pGB2 [18] and pKD3 [16], a gift from F. Boccard laboratory. pGBKD3-2-Pr_dnaB_-Venus-linker (JJC6182) was constructed by PCR ligation and carries the *venus* gene sequence [19] attached in 3’ to the 11 amino acids linker sequence used in R. Reyes-Lamothe et al [15], and expressed under the control of the *dnaB* promoter. PCR amplification from this plasmid was used for the construction of a *venus*-*dnaB* Nter fusion inserted at the endogenous locus (JJC6185). This strain and the pGBKD3-2-Pr_dnaB_-Venus-linker plasmid were used for N-ter fusion constructions.

#### Venus-recO

*recO* is the only non-essential gene in the *rnc-era-recO-pdxJ* operon. Nevertheless, as described below, we could insert the pKD3 Cm^R^ gene together with the *venus*-linker sequence upstream of chromosome *recO* gene without loss of viability or increase in UV irradiation sensitivity (S1B Fig). In a first step a pKD3-Venus-linker plasmid (JJC6214) was constructed by cloning into the *Bst*B1-*Bmg*B1 sites of pKD3 the *venus* gene sequence followed by the 11 amino acids linker sequence and amplified from the chromosomal *venus-dnaB* gene fusion. The following oligonucleotides were used for PCR amplification: 5’ TA GAT GCT TAT TCG AAA AGA GTA ACT CCG ATG CCA AAG AAG AGC AAG GG 3’ and 5’ TAG ATG CTT ACA CGT CGA ATT CGC CAG AAC CAG CAG 3’. On the PCR product, *venus* (ATG in bold) is preceded by the *recO* RBS sequence (in bold), and is attached by its C-terminus to the 11 amino acids linker. In a second step, the pKD3-Venus-linker sequence was amplified by PCR using a forward oligonucleotide carrying the last 50 nucleotides (NT) of *era* followed by the pKD3-Venus-linker sequence downstream of the Cm^R^ gene: 5’ CCG ACG ACG AAC GCG CAC TGC GCA GTC TCG GTT ACG TTG ACG ATC TTT AAT ATG AAT ATC CTC CTT AGT TCC 3’, and a reverse oligonucleotide carrying the first 50 nucleotides of *recO* followed by the 20 last nucleotides of the 11 amino acids linker: 5’ TCGC TCC ACG GGC GAC TAT GCA GGA CAA ATG CGC GCT GCC AGC CT TCC ATG AAT TCG CCA GAA CCA GCAG 3’. The PCR product carries: the last 50 NT of *era* gene, the pKD3 Cm^R^ gene, *venus* downstream of the *recO* RBS, the 11 amino acids linker, and finally the 50 first NT of *recO*. Transformation by electroporation of DY330 [17] creates a strain in which the *venus-recO* fusion is inserted at the endogenous locus, adjacent to a Cm^R^ gene. The fusion was verified by sequencing and P1 transduced in MG1655, selecting Cm^R^. The resulting strain, JJC6229, was verified by PCR and used for microscopy. JJC6229 showed 100% plating efficiency on minimal medium and LB at 30°C, 37°C or 42°C (S1B Fig). As *recO* is essential for gap repair, we check that the *venus-recO* fusion is functional by measuring survival to UV irradiation. As shown in S1B Fig the presence of the fusion did not affect survival to UV irradiation.

#### Venus-recG

*recG* is the last gene of the *rpoZ-spoT-trmH-recG* operon. In a first step, a DY330 derivative deleted for the entire *recG* sequence was constructed (JJC6184). For this purpose, the Kan^R^ gene of pKD4 [16] was amplified by PCR using the following oligonucleotides: 5’ GAA GCT GAT GCC GAC TGG TGG GCT ACT ATG CAG GCT GCA GGG TAA GTG CC TGT GTA GGC TGG AGC TGC CTT C 3’ and 5’ ATC CGG CAG GAA GGT AGG GTA ACC TGA AAT GGC GGT CTT CTC ACT GCC GCC ATA TGA ATA TCC TCC TTA 3’. On the PCR product, the Kan^R^ gene is flanked by 50 nucleotides upstream and downstream of *recG*, allowing a complete deletion of the gene. In a second step, the *recG* coding sequence was cloned in pGBKD3-2, using the oligonucleotides: 5’ GAC GTT CCC GGG ATG AAA GGT CGC CTG TTA GA3’ and 5’ CTG CAG GTC GAC GCC TGA TAC GCT TCG CTT ATC 3’ (JJC6202, pGBKD3-2-recG). In a third step, a PCR ligation was used to synthesize a linear DNA fragment allowing replacement of Δ*recG*::Kan^R^ sequence of JJC6184 by a *venus*-linker-*recG*-Cm^R^ sequence. (i) PCR1 amplified *venus-linker* from pGBKD3-2-Pr_rep_-venus, flanked by the 50 last NT of *trmH* and the 30 first nucleotides of *recG*, using the following oligonucleotides: GAA GCT GAT GCC GAC TGG TGG GC TAC TAT GCA GGC TGC AGG GTA AGT GCC ATG CCA AAG AAG AGC AAG GGC GAG GAG CTG T and 5’ ATC TAA CAG GCG ACC TTT CAT GAA TTC GCC AGA ACC AGC AGC GGA GCC AGC CGA 3’. (ii) PCR2 amplified *recG* and Cm^R^ from pGBKD3-2-recG using the following oligonucleotides: 5’ TC GGC TGG CTC CGC TGC TGG TTC TGG CGA ATT C ATG AAA GGT CGC CTG TTA GA 3’ and 5’ ATC CGG CAG GAA GGT AGG GTA ACC TGA AAT GGC GGT CTT CTC ACT GCC GC GTG TAG GCT GGA GCT GCT TC 3’. In the PCR product, the *recG* gene is preceded by the 11 amino acids linker, and the Cm^R^ gene is followed by 50 NT of the chromosomal sequence after *recG*. (iii) A ligation by PCR was realized to link the products of PCR1 and PCR2, which can pair by the linker sequence, using the forward oligonucleotide of PCR1 and the reverse oligonucleotide of PCR2. The final PCR product was used to transform DY330-Δ*recG*::Kan^R^ (JJC6184) by electroporation, creating a strain in which the *venus-recG* fusion is inserted at the endogenous locus, adjacent to a downstream Cm^R^ gene (JJC6247). The fusion was verified by sequencing and P1 transduced in MG1655, selecting Cm^R^. The resulting strain, JJC6255, was verified by PCR and used for microscopy. JJC6255 showed 100% plating efficiency on minimal medium and LB at 30°C, 37°C or 42°C (S1B Fig). *recG* inactivation confers a high sensitivity to UV irradiation when introduced in a *ruvAB* or a *ruvC* context. The activity of the *venus-recG* fusion was verified by combining it with a *ruvA*::Tn*10* mutation (JJC6262) and measuring survival to UV irradiation. As shown in S1B Fig the presence of the fusion did not modify survival of the *ruvAB* mutant to UV irradiation, showing that the *venus-recG* chromosomal fusion is fully functional.

#### Pr_recG_-*venus*

The pKD4 Kan^R^ gene [16] was amplified using a forward oligonucleotide composed of the 50 last nucleotides of *venus*, two stop codons and the 20 first nucleotides of KanR: 5’ AG TTC GTG ACC GCC GCC GGG ATC ACT CTC GGC ATG GAC GAG CTG TAC AAG TAA TGA AAC TTC AAG ATC CCC TCA CG 3’and a reverse oligonucleotide composed of 50 NT after the *recG* sequence and the 20 last nucleotides of Kan^R^: 5’AT CCG GCA GGA AGG TAG GGT AAC CTG AAA TGG CGG TCT TCT CAC TGC CGC AGA GCG CTT TTG AAG CTG GG 3’. Introduction of the PCR fragment in the JJC6247 chromosome replaced the *recG* coding sequence of the *venus-recG* fusion by two stop codons and the adjacent Cm^R^ by a Kan^R^ marker (JJC6442). The *venus* gene was checked by sequencing in JJC6442. The *rpoZ-spoT-trmH-venus* operon was then P1 transduced to MG1655 using the adjacent Kan^R^ marker (JJC6452), and checked by PCR. As expected this *recG* mutant strain was fully viable (S1B Fig).

#### Venus-priA

In a first step the *priA* promoter region was amplified by PCR from MG1655 chromosome using the following oligonucleotides; 5’ CCA GCT TGA CAC GTC GAC AAT CAT ACA GAA ATT AAC CAG CG 3’ and 5’ CCT CAC GCT TGC TCT TCT TTG G CAT AGC ATC ATC CTG ACT TG 3’. In the PCR product, the *priA* promoter is flanked by *Sal*1 and *Sap*1 restriction sites that were used to replace the *dnaB* promoter in pGBKD3-2-Pr_dnaB_-Venus-linker by the *priA* promoter, yielding pGBKD3-2-Pr_priA_-Venus-linker (JJC6246). The *priA* promoter region in this plasmid was verified by sequencing. In a second step the plasmid was used for PCR amplification using (i) a forward oligonucleotide composed of 50 NT of the MG1655 chromosome before the *priA* promoter followed by 20 NT before the FRT site of the Cm^R^ gene on the plasmid: 5’ AA ATT AAC CAG CGT ATG CAA ACT GAT CCG CAC TCT TCT ACG GCA ATG TGT GTG TAG GCT GGA GCT GCT TC 3’, and (ii) a reverse oligonucleotide composed of the first 50 NT of the *priA* coding sequence followed by the end of the 11 amino acids linker: 5’ TCA AAG GTA CGA GGA AGC GGA ACG GGC AAG GCA ACG TGG GCA ACG GGC ATG AAT TCG CCA GAA CCA GCA GCG 3’. The resulting PCR product carries 50 NT upstream of *priA*, the Cm^R^ gene, *venus* under the control of the *priA* promoter attached by the linker to the first 50 NT of the *priA* coding sequence. Transformation of DY330 by electroporation produced a strain in which the *venus-priA* N-ter fusion is adjacent to an upstream Cm^R^ gene (JJC6259). The fusion was verified by sequencing and P1 transduced, selecting Cm^R^, into MG1655. The resulting strain, JJC6269, was verified by PCR and used for microscopy. The *priA* null mutant is sensitive to rich medium and to UV irradiation [20]. JJC6269 showed 100% plating efficiency on minimal medium and LB at 30°C, 37°C or 42°C (S1B Fig), and was as resistant to UV irradiation as the wild-type strain on minimal medium (S1B Fig), indicating that the *venus-priA* fusion is fully functional in our experimental conditions (minimal medium, 30°C).

#### Venus-rep

In a first step the *rep* promoter was cloned in pGBKD3-2-Pr_dnaB_-Venus-linker replacing the *dnaB* promoter upstream of *Venus* by the *rep* promoter (pGBKD3-Pr_rep_-Venus-linker, JJC6195). For this purpose, the following oligonucleotides were used to amplify the *rep* promoter from MG1655 chromosome: 5’ CCA GCT TGA CAC GTC GAC CAG CCA ACC GGT TAG CG GCT 3’ and 5’ CCT CAC GCT TGC TCT TCT TTG GCA TAG GTG TAT TGC TCA ATCT 3’. In the PCR product, the *rep* promoter is flanked by *Sal*1 and *Sap*1 restriction enzymes which were used to replace the *dnaB* promoter in pGBKD3-2-Pr_dnaB_-Venus-linker. The *rep* promoter region in pGBKD3-2-Pr_rep_-Venus-linker was verified by sequencing. In a second step the plasmid was used for PCR amplification using (i) a forward oligonucleotide composed of 50 NT of the MG1655 chromosome before the *rep* promoter followed by 20 NT before the FRT site of the Cm^R^ gene on the plasmid: 5’ TCT AAT GGA TTC ACG ATG AAC TCC GAT TTC GGT CTT CTC TCT CTG ATT TA GTG TAG GCT GGA GCT GCT TC 3’, and (ii) a reverse oligonucleotide composed of the first 50 NT of the *rep* coding sequence followed by the end of the 11 amino acids linker: 5’ GGG CCG GTA ACG AAT TCG ACA GCT TGT TGT TGG CCG GGG TTT AGA CGC AT G AAT TCG CCA GAA CCA GCA GCG 3’. The resulting PCR product carries 50 NT upstream of *rep*, the Cm^R^ gene, *venus* under the control of the *rep* promoter attached by the linker to the first 50 NT of the *rep* coding sequence. Transformation of DY330 by electroporation produced a strain in which the *venus-rep* N-ter fusion is adjacent to an upstream Cm^R^ gene (JJC6210). The fusion was verified by sequencing and P1 transduced, selecting Cm^R^, into MG1655. The resulting strain, JJC6220, was verified by PCR and used for microscopy. JJC6220 showed 100% plating efficiency on minimal medium and LB at 30°C, 37°C or 42°C (S1A Fig). As inactivating *rep* is co-lethal with *uvrD* mutation [21], the activity of the *venus-rep* fusion was tested by combining these two mutations. *uvrD venus-rep* (JJC6226) strain was constructed by P1 transduction with the expected efficiency and was fully viable (S1A Fig).

#### Pr_rep_-*venus*

The Cm^R^ in JJC6220 was excised by Flp recombination yielding JJC6236. The pKD3 Cm^R^ gene was then amplified using a forward oligonucleotide composed of the 50 last nucleotides of *venus*, two stop codons and the 20 first nucleotides of the Cm^R^ gene: 5’ AG TTC GTG ACC GCC GCC GGG ATC ACT CTC GGC ATG GAC GAG CTG TAC AAG TAA TGA CGG AAG ATC ACT TCG CA 3’, and a reverse oligonucleotide composed of the last 50 NT of *rep* followed by the 20 last nucleotides of Cm^R^: 5’ AT GAG TAA GTG CCG GAT GCG ATG CTG ACG CAT CTT TTC CGG CCT TGA TTA CCT GCC ACT CAT CGC AGTA 3’. Electroporation of JJC6263 with the PCR fragment replaced the *rep* gene in the *venus-rep* fusion by two stop codons, and introduced an adjacent downstream Cm^R^ gene (JJC6405). Pr_rep_-*venus* was checked by sequencing and was co-transduced with Cm^R^ into MG1655, yielding JJC6413, which was checked by PCR. As any *rep* mutant, the strain was fully viable (S1A Fig)

### Bacteria microchamber

#### 1. Microchamber

**Fig 1:**
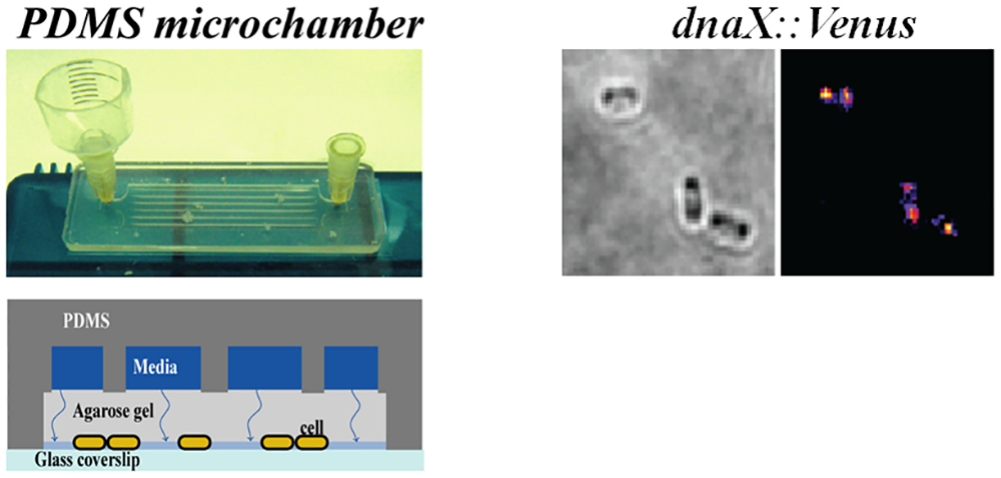
Assembly of bacterial microchamber. A) Picture of an assembled microchamber connected to a reservoir at one side and to a needle at the other side, which will be connected later to a syringe pump. B) Schematic of a cross section of the microchamber. C) Image of bacteria growing in 2 dimensions.

A PDMS chamber is obtained by mixing reagents from the Sylgard 184 Silicone Elastomer Kit as specified from the manufacturer (10:1 elastomer: curing agent). The silicon mix is poured onto a microchamber mold and incubated at 100°C for 45 min to 1 hr. Microchambers are then removed from the mold and covered with a glass coverslip previously incubated in plasma cleaner for 5 min to clean the surface. A 1–2% gel pad is prepared by mixing M9 glucose media with agarose. When melted, ~500 μl of this M9-agarose gel is poured on a mold to obtain an agarose gel pad 400 μm thick. 5 μl of a 1/100 dilution in fresh M9 glucose of an overnight bacterial culture at 30°C are placed on the clean glass coverslip. The drop of bacterial suspension is covered with the agarose gel pad and incubated for 3–5 min at room temperature (RT). During this time, silicon glue (elastosil E41 from Wacker) is applied on the PDMS chamber which is clamped on the assembled glass/bacteria/gel pad and is kept at RT for 40 min-1 hr. After this time, using a needle one side of the microchamber is connected to a reservoir, which is used to introduce the M9 0.4% glucose, 0.006% casamino acids, media (Fig 1). The other side of the microchamber is connected to a syringe pump, which is used to flow the media between the PDMS microchamber and the gel pad. The M9 glucose media reaches the bacteria by simple diffusion through the agarose gel pad. The microscope is equipped with a thermo-regulated sample holder. Cells are placed on the microscope stage at 30°C for 90–120 min and observed to ensure growth and to take control pictures of growing cells. Fluorescence images of group of bacteria are then taken every 1 to 2 min for 40 min.

For experiments at 42°C the microchamber is placed in a 30°C incubator while the sample holder is shifted to 42°C. Once the sample holder temperature reaches 42°C, the microchamber is placed back and pictures of group of bacteria are taken every 30 min for 2 hours.

#### 2. Bacterial growth rate determination and foci counting in microchamber

For bacterial growth rate determinations, images of bacteria growing at 30°C in presence of M9 0.4% glucose 0.006% casamino acids flowing in the microchamber were collected every 30 minutes. The number of bacteria in a given field was counted for 4 to 6 hours and growth curves were fitted with a single exponential to measure the average doubling time in our experimental conditions. Results were similar for all strains (Table 1).

**Table 1:** Generation times in the microchamber at 30°C.

strain	Fluorescent protein	Generation time (min)
JJC1392	None	71 / 84
JJC6633	*holA*-venus	100 / 68 / 83 / 98 / 68 / 98 / 97
JJC6632	*dnaX*-venus	71 / 90
JJC5956	*dnaX*-YPet	61 / 71 / 107
JJC5349	*holD*-Ypet	61 / 72
JJC6229	Venus-*recO*	68 / 70
JJC6255	Venus-*recG*	62 / 66
JJC6452	P_recG_-venus	70 / 74 / 75
JJC6269	Venus-*priA*	70 / 73 / 76
JJC6220	Venus-*rep*	68 / 74
JJC6413	P_rep_-venus	62 / 70

To count focus containing cells in exponential phase, bacteria were placed on the agarose pad and grown for 1.5 to 2.5 hours in the microchamber at 30°C with constant medium feeding. First bacteria in a microscopy field were observed to check that they were dividing. Then, changing observation field for each image, a brightfield image and a series of laser illuminated images (one very 200 ms) were taken, for up to about 15–20 different fields, i.e. ~200–250 bacteria per experiment. Images were analyzed as described below. To count cells containing foci in stationary phase, 24 hours cultures were washed three times in minimum medium salt solution, bacteria were placed on the agarose pad and minimum medium salt solution was flowed in the microchamber. Bacteria in a microscopy field were observed for 1.5 to 2.5 hours at 30°C to check that they were not growing and not dividing. Cell sizes were measured and, as expected, stationary phase cells were smaller than exponentially growing cells. Bacterial images and focus images were taken and analyzed as for exponentially growing cells.

### Microscope set-up

The microscope system is composed of an Olympus PlanApo 100X /1.40 oil immersion objective, a Pifoc (PolytecPI) to adjust the focus, a custom-made objective holder that is thermo-regulated using a Peltier system (from 20°C to 45°C) (Wavelength Electronics), a cooled EMCCD camera IXON Ultra (Andor technology) used for fluorescence detection and bacteria imaging. A white LED (Thorlabs) was used to visualize bacteria and turned off during fluorescence measurements. A laser beam at 515 nm (Cobolt Fandango 50TM 515nm) was used to excite fluorophores. Laser beam was separated from the YPet/Venus emission using a dichroic mirror (Di01-R442/514/561 Semrock). The YPet and the Venus emission was filtered using an emission filter (FF01-485/537/627 Semrock). To reduce photobleaching we used a stroboscopic illumination: an acousto-optical modulator (AOTNFnC-VIS-TN 1001 from AA Opto-Electronics) was placed in the laser beam path as a shutter (S2 Fig). The acousto-optical modulator was synchronized with the CCD camera frame rate using as trigger the camera fire signal through a custom-made electronic circuit. Acquisition frequency and stroboscopic illumination were controlled by the camera acquisition software (Andor Solis). Time between frames was 200 ms, with 40 ms exposure time at ~2.5 W/cm^2^.

### Image analysis

Images were analyzed manually. To avoid human bias, for all strain/condition each of the four (or three) independent experiments was analyzed by a different experimentalist, so that results are the average of manual analysis by four (or 3, when indicated) different people.

**Fig 2:**
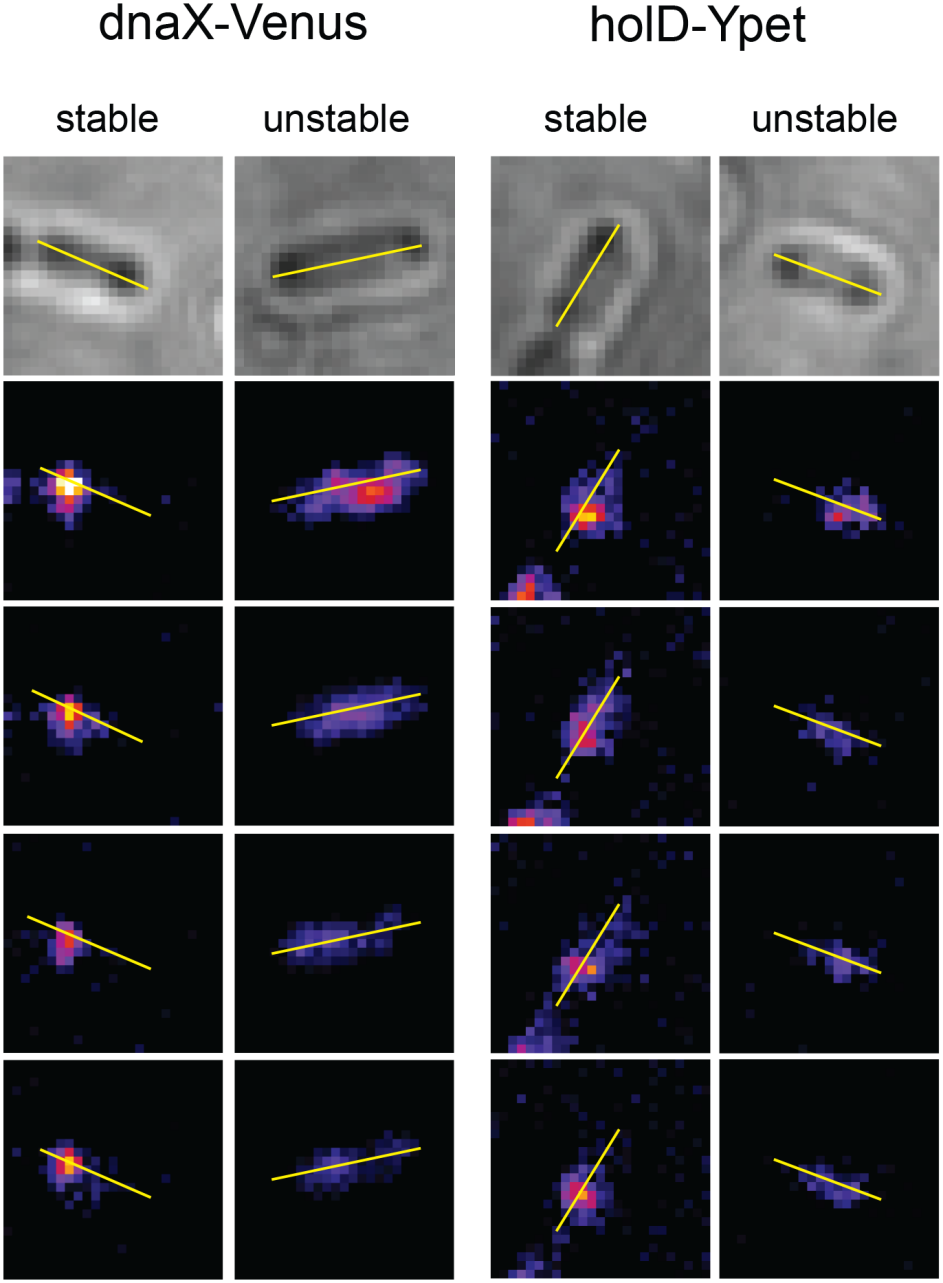
Examples of stable and unstable replisome proteins foci. Using the Image J software, a line was drawn from pole to pole on each cell on the brightfield image (top picture), and translated to the fluorescent image frames (four bottom pictures show the first four frames, used for the analysis). This line is used as a fixed mark and indicates the position of the bacteria on the fluorescent images, where the general shape of bacteria is not visible. Spots were manually detected, only spots counting more than 5 pixels (I pixel ~ 140 nm) were taken into account as spots counting less than 5 pixels could be observed at a relatively high rate in wild-type cells devoid of fluorescent protein and presumably result from autofluorescence. As shown in S4 Fig, for foci with a maximum intensity on the line in the first fluorescent picture, the intensity along the line was used to determine whether a focus is stable (moves by no more than one pixel on at least 3 of the first 4 frames), or unstable (moves by more than one pixel or disappears). For other foci stability was determined by eye, using the pole to pole line as a position reference.

**Fig 3:**
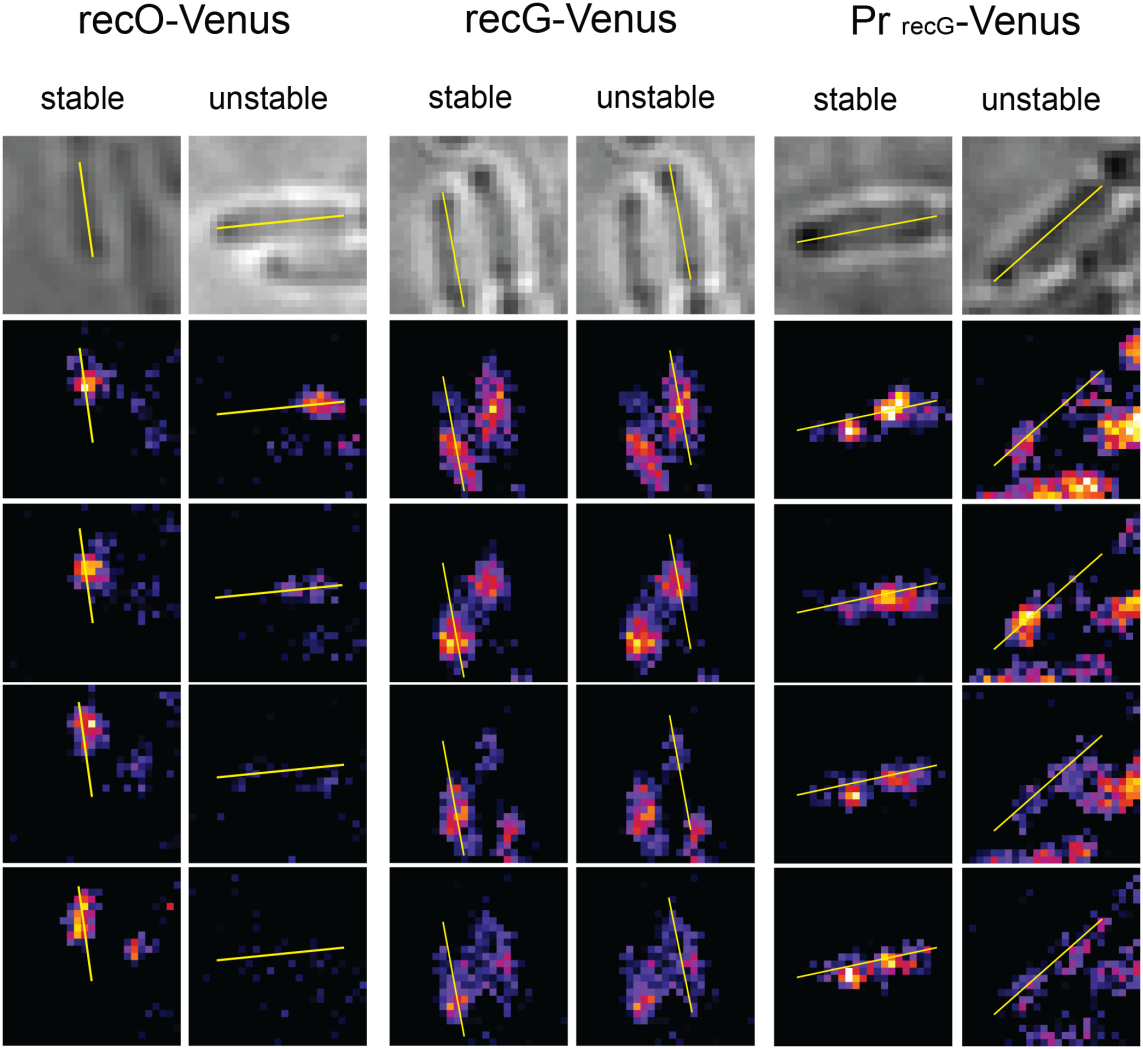
Examples of stable and unstable RecO-Venus and RecG-Venus foci, and of stable and unstable foci in cells that express Venus from the *recG* promoter. For each bacteria, the first four frames of the fluorescent series of images are shown below the brightfield picture (top). See the legend to Fig 2 for details.

**Fig 4:**
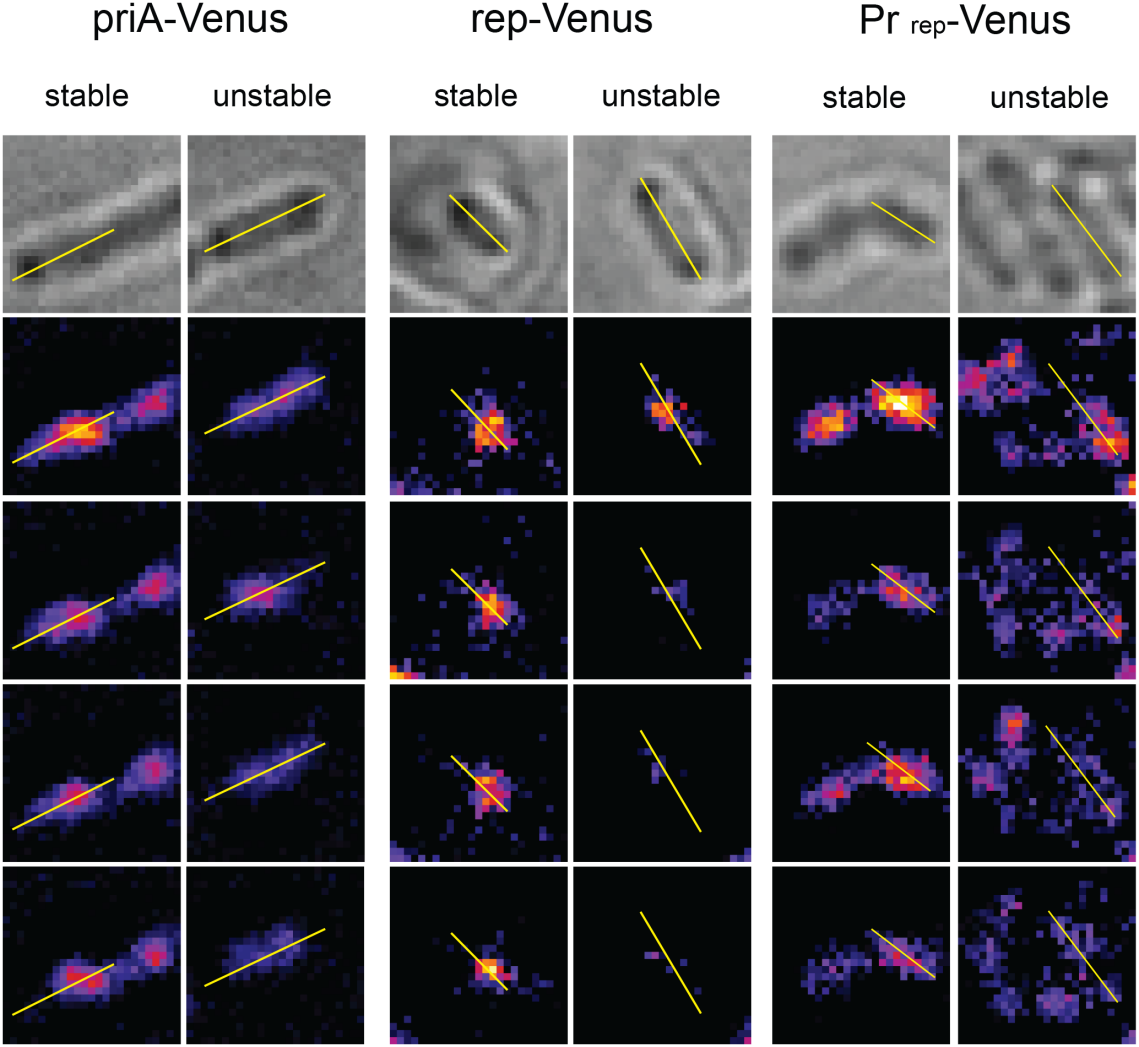
Examples of stable and unstable PriA-Venus and Rep-Venus foci, and of stable and unstable foci observed in cells that express Venus from the *rep* promoter. For each bacteria, the first four frames of the fluorescent series of images are shown below the brightfield picture (top). See the legend to Fig 2 for details.

Fluorescence images were first treated with a custom-made Matlab program to compensate unequal illumination assuming a Gaussian profile illumination. Image treatments and analysis were then done using the open-source software ImageJ. Images were treated to remove background and thus allow better foci detection (S3 Fig). For this purpose, fluorescence images were duplicated and treated using the ImageJ’s plugin Filter “Sigma Filter Plus”. This plugin-Filter provides a selective mean (averaging) filter. The filter smooths an image by taking an average over the neighbouring pixels, but only includes those pixels that have a value not deviating from the current pixel by more than a given range. One of the duplicated images was treated using the plugin filter parameters: Radius = 10 pixels, using pixels within 5 sigma range and Minimum Pixel Fraction = 0.2. The other fluorescent image was not treated, and the two images were subtracted to create an image with only the fluorescence spots (Figs 1C, 2, 3 and 4, and S3 Fig). Foci were identified manually as groups of at least 5 pixels, Their position coordinates were determined by finding the position of spot fluorescence intensity maxima. A line was drawn on each bacterium on the brightfield image from pole to pole and translated on the treated fluorescent images. This line was used as a fixed mark to compare foci position, defined by the position of the maximum intensity pixel(s), in the four analyzed frames. Additionally, for foci that were centered in the longitudinal axis, intensity along the line was used to determine their stability (S4 Fig). The first four frames (800 ms) were analyzed; foci were called stable when they remained immobile or moved less than one pixel for at least 3 frames (authorizing one blinking event) and called unstable when they moved for more than 1 pixels or disappeared (see examples in Figs 2, 3 and 4, and S4 Fig). Foci were called central when located between 0.4 and 0.5, and lateral when located between 0.15 and 0.33 of cell length. Nearly no foci were polar (<0.15) and foci between 0.33 and 0.4 were considered as neither central nor lateral.

## Results

### Microscopy set-up and fluorescent proteins used in this work

A single fluorescent protein can be detected in living *E*. *coli* cells [3, 14, 22–27]. We implemented a version of the detection by localization technique where the image acquisition time is large enough to average the fluorescence of freely diffusing proteins over a large volume, producing only a background noise, allowing the detection of only slowly diffusing proteins where this averaging does not occur and that appear as bright spots [22, 23]. We used stroboscopic illumination, which, by limiting illumination duration, is supposed to reduce photobleaching (see Material and Methods). In our conditions (40 ms exposure time), proteins involved in replication and fused to a fluorescent protein should diffuse more slowly when bound to the replisome than fluorescent molecules that do not bind DNA and diffuse freely.

The yellow fluorescent protein Venus was fused to proteins of interest (see Material and Methods), and previously reported DnaX-YPet and HolD-YPet fluorescent fusions were used as controls [15]. YPet derives from Venus by mutations that increase CyPet-YPet FRET and the two fluorescent proteins show a similar brightness [28], and a similar behavior in our microscope (see below). All fluorescent fusions used in this work were fully functional and all strains showed a generation time of 70–80 min in our experimental conditions (S1 Fig, Table 1). DnaX-YPet and HolD-YPet were previously shown to be present in 3 and 4 copies per replisomes, respectively [3, 14]. Here, reproducible quantification of the number of fluorescent molecules per focus was unsuccessful and only the proportions of cells showing one or two foci were determined.

### Our microscope allows detection of foci containing three or four molecules, but does not allow single molecules detection

For microscopy observations cells were grown in a microchamber fueled with minimal medium 0.4% glucose 0.006% casamino acids at 30°C (Fig 1, cf Materials and Methods). DnaX-Venus, DnaX-YPet and HolD-YPet foci were observed in 77.6%, 86% and 78.5% of growing cells (Table 2, Fig 2). However, we observed that some foci were short-lived while others were more stable, and classified foci in two categories. Foci called “stable” remain at the same position (± 1 pixel, about 120–130 nm per pixel) in at least three of the first four frames (40 ms illumination, 160 ms between illuminations, one frame every 200 ms). Unstable foci were not at the same position in three of the first four frames, either because they shifted for 2 pixels or more, or because they disappeared (see examples of stable and unstable foci in Fig 2 and S4 Fig). The 77.6% of cells with at least one DnaX-Venus focus thus correspond to 53% containing at least one stable focus and 24.6% with only an unstable one. Similarly, 61.5% and 54.5% cells showed at least one stable focus and about 24% only an unstable one with DnaX-YPet and HolD-YPet, respectively (Table 2).

**Table 2:** Proportion of stable and unstable foci with different protein fusions.

Strain	Fluorescent protein	Proportion of cells with foci (%)				N^a^
		0	1 stable	2 stable	unstable	
JJC1392	none	98.5 ± 0.3	0.6 ± 0.2	<0.2	1 ± 0.4	584 (2)
JJC6633	*holA*-venus Expo	41.8 ± 5.6	24.9 ± 5.4	1.7 ± 1.3	31.6 ± 5.1	1514 (6)
JJC6632	*dnaX*-venus Expo	22.4 ± 2.1	49.4 ± 1.7	3.6 ± 1.5	24.7 ± 2.8	1033 (4)
	*dnaX*-venus Stat	45.3 ± 3.8	24.8 ± 2.6	2.0 ± 0.8	27.8 ± 3.1	889 (4)
JJC5956	*dnaX*-YPet Expo	14.2 ± 3.3	54.3 ± 3.1	7.2 ± 3.8	24.5 ± 4.4	1059 (4)
	*dnaX*-YPet Stat	38.3 ± 1.4	25.7 ± 2.3	0.8 ± 0.9	35.3 ± 1.4	1021 (4)
JJC5349	*holD*-YPet Expo	21.5 ± 4.8	49.8 ± 4	4.5 ± 2.8	24.2 ± 5.8	1048 (4)
	*holD*-YPet Stat	37.4 ± 3	23.6 ± 2.9	0.7 ± 0.4	38.3 ± 1.4	989 (4)
JJC6229	venus-*recO* Expo	59.5 ± 4.1	8.5 ± 3	0.5 ± 0.5	31.4 ± 2.9	843 (3)
JJC6255	venus-*recG* Expo	18.2 ± 4.4	34 ± 2	3.9 ± 3.2	43.9 ± 7.6	1261 (5)
	venus-*recG* Stat	10.7 ± 3.1	43.1 ± 1.2	4.4 ± 1.6	41.8 ± 3	976 (4)
JJC6452	Pr_recG_-venus Expo	18.3 ± 1.8	31.4 ± 2.8	2 ± 1.9	48.3 ± 2.1	1025 (4)
JJC6269	venus-*priA* Expo	15 ± 2.9	49.7 ± 4.9	8.4 ± 4.4	27 ± 3.5	689 (3)
	venus-*priA* Stat	11.6 ± 3.8	50.7 ± 3.7	5.9 ± 0.9	31.9 ± 5.3	1055 (4)
JJC6510	venus-*priA recA* p-RnaseH Stat	9.8 ± 1.7	56.1 ± 3.3	7.3 ± 2.5	26.8 ± 4.7	807 (4)
JJC6220	venus-*rep* Expo	26.1 ± 1.6	32.4 ± 4	4 ± 2.6	37.5 ± 4.8	911 (4)
	venus-*rep* Stat	28.4 ± 5.1	22.5 ± 4.5	0.7 ± 1	48.4 ± 4.2	949 (4)
JJC6413	Pr_rep_-venus Expo	25.8 ± 2.8	24.8 ± 6.1	5 ± 3.2	44.4 ± 3.9	1269 (4)

^a^: N is the number of cells analysed, and between parenthesis the number of independent experiments.

“Expo” stands for cells in exponential growth. “Stat” stands for stationary phase cells.

In growing *E*. *coli* cells, a unique central replication focus is observed when replisomes co-localize after replication initiation and during replication termination, and two lateral foci appear after replisome splitting, during replication of the two chromosome arms. Depending on growth conditions, the proportion of cells with a unique central focus was reported to be lower or equal to the proportion of cells with two lateral foci [15, 29]. In our experiments the proportion of growing cells with two foci was lower than expected (Table 2) suggesting that we do not detect all replisomes.

To ascertain that stable foci of DnaX-Venus, DnaX-YPet and HolD-YPet actually correspond to progressing replisomes, we tested whether they are specific for cell growth conditions. This question could theoretically be addressed using a mutant thermosensitive for replication. However, the number of stable foci decreased in wild-type cells shifted to 42°C, indicating that fluorescent proteins are thermosensitive (S5 Fig). Therefore, stationary phase cells incubated in salt solution were used as non-replicating controls (cf Materials and Methods). The proportion of cells showing at least one DnaX-Venus, DnaX-YPet or HolD-YPet stable focus dropped from 53–61% to 24–27% in non growing cells (Table 2). This result indicates that the high level of stable foci in exponential phase results from the presence of active replisomes. As DnaX-Ypet and DnaX-Venus behave similarly we conclude that both fluorophores afford the same sensitivity of detection in our microscopy assay.

Active replisomes have been shown to be positioned at midcell when both replication forks colocalize (after replication initiation and prior to termination) and at positions ¼ ¾ after replisome segregation [15, 29, 30]. As shown in Table 3, a large proportion of single foci were lateral, indicating that the lack of cells with two foci results from one replisome only being visible after segregation to the ¼ ¾ positions. However, focus localization could not be used here as an indicator of replisome function because the position of DnaX-Venus, DnaX-YPet and HolD-Ypet foci was not significantly different in exponential and stationary phase (Table 3).

**Table 3:** Stable foci positions.

Strain	Fluorescent protein	Proportion of cells with foci (%)			N^a^
		0.15–0.33 Lateral	0.4–0.5 Central	0.33–0.40 Neither	
Expected from random positioning on the nucleoid (0.15–0.5)^b^		(18/35) x 100 51.4	(10/35) x 100 28.6	(7/35) x 100 20	
JJC6632	*dnaX*-venus Expo	60.3 ± 6.6	36.6 ± 5.4	1.7 ± 3.9	476
JJC6632	*dnaX*-venus Stat	68.3 ± 8.1	30.4 ± 9.4	1.4 ± 1.2	221
JJC5956	*dnaX*-YPet Expo	38.3 ± 12.4	51.4 ± 10	10 ± 7.4	574
JJC5956	*dnaX*-YPet Stat	34.8 ± 15.1	53.5 ± 11.1	11.8 ± 6	262
JJC5349	*holD*-YPet Expo	38.7 ± 12.5	48.5 ± 8	12.8 ± 7.9	522
JJC5349	*holD*-YPet Stat	48.9 ± 15.1	42.4 ± 5.5	8.6 ± 8.2	233
JJC6255	venus-*recG* Expo	44.5 ± 9.8	41.7 ± 3.1	13.7 ± 14.5	429
JJC6255	venus-*recG* Stat	44.4 ± 9.7	46.3 ± 5.7	9.3 ± 6	421
JJC6452	Pr_recG_-venus Expo	50.7 ± 11.2	34.5 ± 7.8	14.8 ± 4	258
JJC6220	venus-*rep* Expo	48.9 ± 8.3	41.8 ± 5.1	9.3 ± 3.2	235
JJC6220	venus-*rep* Stat	44.4 ± 3.1	44.9 ± 2.7	10.8 ± 4.2	214

^a^: N is the number of unique stable foci used for ratio determinations.

^b^: Foci positions were determined by dividing cells in four regions:

-central between 0.4 and 0.5 (region length 0.1)

-lateral between 0.15 and 0.33 (region length 0.18)

-neither central nor lateral between 0.33 and 0.4 (region length 0.07)

-polar between 0 and 0.15 (region length 0.15)

Given that polar foci were extremely rare with all strains, the ratio expected from random positioning in the three remaining regions was calculated by dividing the central, lateral or neither region lengths by the sum of these three region lengths, 0.35.

“Expo” stands for cells in exponential growth. “Stat” stands for stationary phase cells.

We constructed a strain with a HolA-Venus fusion expressed from the endogenous locus (contrary to DnaX and HolD, HolA is present in a single copy at the replisome). As for all fusion proteins used in this work, the *holA-Venus* gene fusion did not affect cell growth in our experimental conditions (S1A Fig, Table 1). Counting foci in 1514 bacteria from 6 independent experiments showed that only 24.9% contained a stable focus (Table 2). As described below, this proportion is not different from that of cells expressing single Venus proteins, which shows that detection of DNA-bound single molecules is not possible with our set-up.

In conclusion, we can detect proteins that are present in the replisome in three copies as stable foci that are present in a high proportion of cells only in growing cells. Unstable foci are also observed, which are present in a high proportion of cells in the absence of chromosome replication and may therefore correspond to the detection of freely diffusing molecules or molecules transiently interacting with DNA. Since a high ratio of stable *versus* unstable foci is characteristic of active replisomes, provided that the fluorescent protein is present in three copies, we used fluorescent fusion of replisome-interacting proteins to test whether at least 3 copies of these proteins are replisome-bound in growing cells.

### RecG and RecO molecules do not travel with replication forks, or do so in less than 3 copies

In order to determine whether proteins that interact with SSB travel with replication forks, fluorescent N-terminal fusion-proteins derivatives of RecO, RecG and PriA, expressed from their endogenous chromosomal promoters were constructed (see Material and Methods).

RecO, together with RecF and RecR, allows RecA binding to SSB-covered ssDNA, thus promoting RecA-filament formation, the first step of recombinational gap repair [31]. RecO interacts with SSB and facilitates RecA binding [9]. As shown in Table 2, very few stable Venus-RecO foci could be observed (see Fig 3 for examples of stable and unstable foci). This result contrast with DnaX-Venus, DnaX-YPet and HolD-YPet results and suggests that RecO does not travel with forks, or does so in less than 3 copies.

RecG is a helicase that acts on a variety of branched DNA structures *in vitro* [32]. *In vivo*, its main role is to prevent unwanted replication by reducing initiation of replication at D-loops or R-loops and by converting 3’ flaps to 5’ flaps [33]; RecG was also proposed to stabilize D-loops during double-strand break repair [34]. *In vitro*, RecG catalyzes Holliday junction branch migration and *recG* mutations strongly decrease homologous recombination efficiency in cells lacking RuvAB, the main Holliday junction branch migration complex [31]. Conventional epifluorescence microscopy allowed the observation GFP-RecG foci in *E*. *coli*, however, these foci were observed only when the fusion protein was over-produced and increasing protein concentration could lead to mis-localization. Actually, studies of various RecG mutations showed no correlation between RecG activity and focus formation [35].

We constructed a Venus-RecG N-ter fusion expressed from the endogenous locus (see Material and Methods). The Venus-RecG fusion was functional (S1A Fig) and did not affect growth (Table 1). Nearly 35% cells expressing the Venus-RecG fusion showed at least one stable focus, less than DnaX-Venus, DnaX-YPet or HolD-Ypet cells (Table 2, see Fig 3 for examples of stable and unstable foci). To determine whether stable Venus-RecG foci reflect RecG-binding to a subset of replication forks we measured the proportion of stationary phase cells with foci. 43% of non-growing Venus-*recG* cells contained at least one stable focus, indicating that the stable Venus-RecG foci observed in exponential phase do not correlate with the presence of active replication forks (Table 2). We constructed a control strain where the RecG moiety of the fusion protein is deleted and Venus is expressed alone under the *recG* transcriptional and translational signals (Pr_recG_-Venus, see Material and Methods). 32% of exponentially growing Pr_recG_-Venus cells contained at least one stable focus, indicating that stable Venus-RecG foci observed in exponential phase do not correlate with the presence of RecG in the fusion protein (Table 2, see Fig 3 for examples of stable and unstable foci). In conclusion, in contrast with DnaX and HolD, RecG does not form replication-associated foci in our experiments, indicating that it does not travel with forks in three or four copies.

### PriA foci do not correlate with replication

PriA is the main protein for replication restart, it recognizes blocked forks and D-loops, where it promotes loading of the replicative helicase DnaB, and, in turn, replication restart [36]. Epifluorescence microscopy showed GFP-PriA foci localized at replication forks position in *Bacillus subtilis*, suggesting that *B*. *subtilis* PriA travels with replication forks [37]. We constructed a Venus-*priA* fusion expressed from the endogenous locus, which was functional in our experimental conditions (see Material and Methods, S1 Fig, Table 1). Slightly more than 50% of Venus-*priA* cells showed at least one stable focus, however, proportions of stable and unstable foci were similar in stationary and exponential phase (Table 2, see Fig 4 for examples of stable and unstable foci). Stable foci in stationary phase could either result from an intrinsic slow diffusion of Venus-PriA fusion proteins *in vivo*, or from the presence of PriA targets in resting cells. In the absence of *oriC*-initiated replication forks, the main PriA targets are D-loops formed by homologous recombination and R-loops resulting from transcription [36]. We constructed a strain that presumably lacks those owing to inactivation of the main recombination enzyme RecA (preventing D-loops formation), and over-expression of the main R-loops nuclease RNaseH from a plasmid (removing R-loops). The Venus-PriA Δ*recA* pGB-RnaseH strain still showed about 50% cells with a stable focus (Table 2). Therefore, the functional Venus-PriA fusion expressed from its endogenous promoter forms stable foci independently of the presence of known PriA targets, possibly resulting from aspecific PriA interactions with DNA, preventing any conclusion to be drawn on the presence of PriA at progressing replication forks.

### Rep does not travel with replication forks, or does so in less than 3 copies

Rep is an accessory replication helicase that removes proteins from the path of replication forks, and more specifically promotes replication across highly transcribed regions in *E*. *coli* [13, 38, 39]. Rep interacts with the DnaB replicative helicase (6 DnaB proteins per replication fork), and it is unknown whether this interaction allows Rep to travel with progressing forks or promotes its binding upon replication arrest. Previously constructed Rep fluorescent fusions did not allow the detection of foci by epifluorescence [13]. We constructed a functional Venus-*rep* gene fusion at the endogenous locus (see Material and Methods, S1 Fig, Table 1). 32% of Venus-*rep* cells showed at least one stable focus and 38% an unstable focus (Table 2, see Fig 4 for examples of stable and unstable foci). This result indicates that Rep does not travel continuously with forks in 3 or more copies.

The proportion of stable Venus-Rep foci was 10% lower in stationary phase than in exponential phase (22% stable foci and 48% unstable foci). To ascertain whether the 10% difference of stable foci between growing and resting cells could result from Rep binding to a subset of blocked forks, we constructed a strain where the Rep moiety of the fusion protein is deleted and Venus alone is expressed from the *rep* promoter (Pr_rep_-Venus, see Material and Methods). The proportion of cells showing stable and unstable foci was not significantly different in cells expressing Venus only compared to cells expressing Venus-Rep (Table 2, see Fig 4 for examples of stable and unstable foci). We conclude that Rep does not travel with replication forks in 3 copies or more. The level of stable foci in Pr_rep_-Venus exponential cells and in Venus-*rep* stationary phase cells precludes a determination of the proportion of Rep-bound blocked forks. We do not know the reasons for this unexpected proportion of cells with a stable focus in exponential cells expressing Venus alone and in stationary phase cells expressing any fluorescent fusion protein (Table 2).

## Conclusions

HolD and DnaX stable foci are specifically detected in more than 50% growing cells in our experiments, while HolA is not. This confirms the presence of at least 3 HolD molecules per fork *in vivo* [3], and indicates that our technique allows us to detect replisome-associated proteins when these are present in at least three copies. Therefore, our results suggest that the RecO, RecG, and Rep proteins either do not travel with most replication forks, or they do so in less than 3 copies.

It has to be noted that during replication the first SSB tetramers that bind ssDNA on the lagging-strand template are also the last to be removed by DNA synthesis, and, conversely, the last incoming SSBs are the first removed. Consequently, SSB tetramers close to the 3’ end of Okazaki fragments bind ssDNA for 1 to 2 seconds while those close the 5’ end are DNA-bound for a much shorter time. If SSB-interacting proteins bind ssDNA-bound SSB tetramers, they will bind preferentially the SSB tetramers that are the closest to the 3’ end of Okazaki fragments. Alternatively, SSB could interact with various proteins in solution and bind ssDNA as a preformed complex [40, 41]. We could detect 8% and 25% of cells with RecO and RecG stable foci, respectively. This is significantly lower than 50% stable foci observed with DnaX and HolD, two Pol III HE subunits present at active replication forks in three and four copies, respectively. These results argue against the presence of three copies of RecO and RecG at progressing replication forks. RecG and RecO proteins are synthesized in low copy number and consequently (i) the turnover of SSB tetramers on Okazaki fragments templates (at most 2 seconds) may be too short for efficient RecG or RecO binding during replication, and (ii) the *in vivo* binding equilibrium between RecG or RecO and free SSB molecules may be too low for a significant ratio of SSB molecules to be complexed with one of these proteins. RecO and RecG are not thought to play a role during replication progression, as RecO acts behind replication forks during gap repair and RecG is thought to act *in vivo* at Holliday junctions, D-loops and R-loops [32]. Slow SSB turnover on ssDNA gaps, D-loops and R-loops may favor RecO-SSB and RecG-SSB interactions on these DNA structures. PriA acts at blocked replication forks but the high level of stable foci in stationary phase even in the absence of known PriA targets, which indicates a slow diffusion of the Venus-PriA fusion protein possibly due to non-specific DNA-PriA interactions, prevents us from drawing any conclusion on the binding of this protein to progressing forks.

The lethality of *rep* combined with *recBC* or *uvrD* mutations suggests that Rep acts at least once per replication cycle [21, 42]. Furthermore, in contrast with SSB DnaB is not thought to be recycled during replication progression, which should facilitate Rep binding to active replication forks. Nevertheless, nearly as many stable foci were detected in exponentially growing cells that express Venus-Rep as in control cells (non-replicating Venus-Rep and replicating Pr_rep_-Venus cells). We conclude that either Rep travels with forks in one or two copies only, or it binds DnaB only after replication arrest, for example because Rep interacts more efficiently with inactive DnaB molecules than with active ones.

## Supporting Information

S1 Fig Venus fusions are functional. (PDF)

S2 Fig Optical set-up. (PDF)

S3 Fig Image treatment (PDF)

S4 Fig Stable and unstable foci. (PDF)

S5 Fig The Y-Pet fluorophore is thermosensitive. (PDF)
