# Supplementary material for: Are the SSB-Interacting Proteins RecO, RecG, PriA and the DnaB-Interacting Protein Rep Bound to Progressing Replication Forks in Escherichia coli?
Source: PLoS One. 2015 Aug 5;10(8):e0134892. doi: 10.1371/journal.pone.0134892 (PMC4526528; doi:10.1371/journal.pone.0134892)
Supplement: S1 Fig — (PDF) [file pone.0134892.s001.pdf]

Are the SSB-interacting proteins RecO, RecG, PriA and the DnaB-interacting protein Rep bound to progressing replication forks in *Escherichia coli*?

Esma Bentchikou¶, Carine Chagneau¶, Emilie Long¶, Mélody Matelot, Jean-François Allemand and Bénédicte Michel*.

Supplementary Figure S1

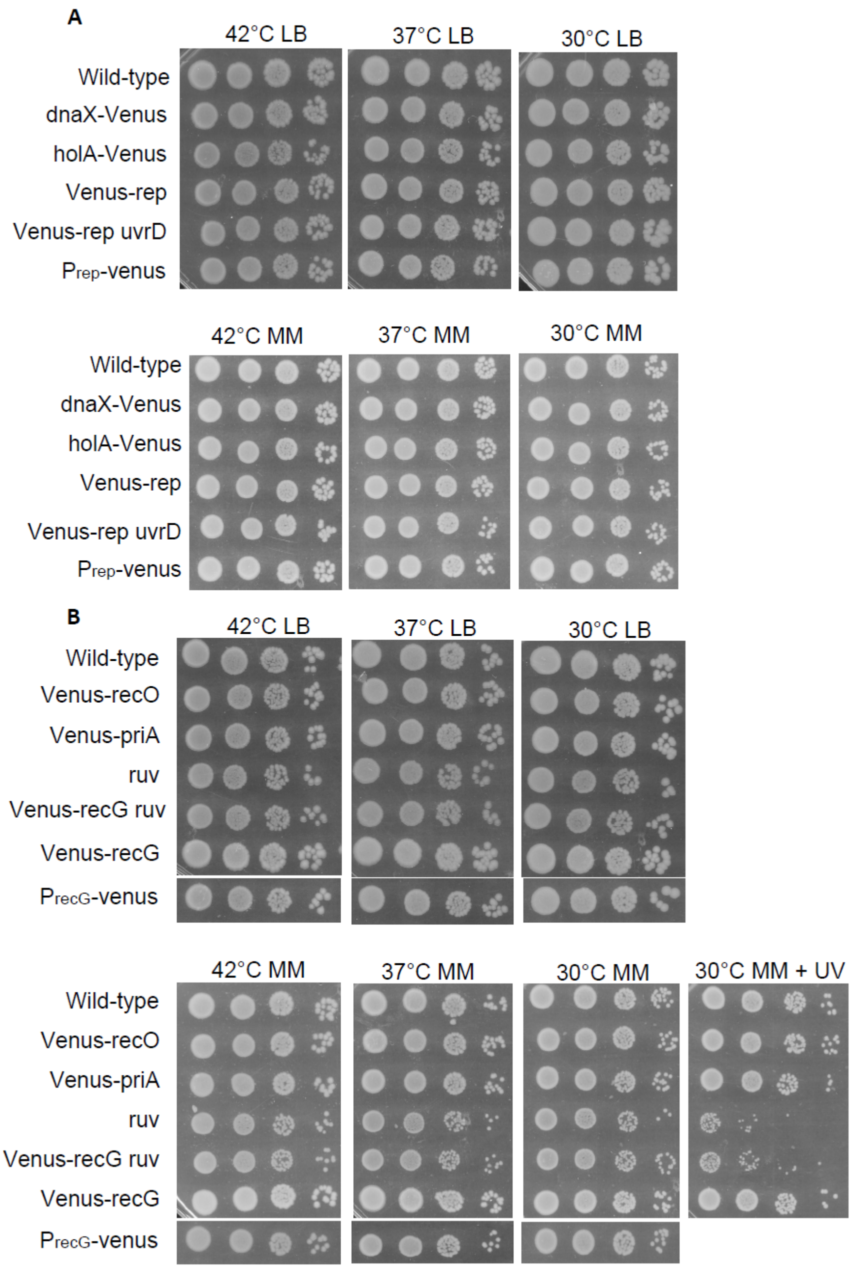

Figure S1. Venus fusions are functional. Isolated colonies grown on MM glucose casaminoacids at 30°C for three days were suspended in 1 ml salt solution and 5 µl drops of 10^{-2} to 10^{-5} dilutions were spotted at 30°C, 37°C, and 42°C on LB (top panels) or MM glucose (bottom panels). (A) From top to bottom: wild-type, *dnaX*-venus (JJC6632), *holA*-venus (JJC6633), venus-*rep* (JJC6220), venus-*rep uvrD* (JJC6226), Pr_{rep}-*venus* (JJCXXXX). (B) From top to bottom: wild-type, venus-*recO* (JJC6229), venus-*priA* (JJC6269), *ruvA60::Tn10* (JJC6261) venus-*recG ruvA60::Tn10* (JJC6262), venus-*recG* (JJC6255), Pr_{recG}-*venus*

(JJC6452). To check that venus-*recO* does not affect survival to UV irradiation in a wild-type context and that venus-*recG* does not affect survival to UV irradiation in a *ruv* context, a MM plate was UV irradiated at 40 joules/m^2 prior to incubation at 30°C.
