# Supplementary material for: Are the SSB-Interacting Proteins RecO, RecG, PriA and the DnaB-Interacting Protein Rep Bound to Progressing Replication Forks in Escherichia coli?
Source: PLoS One. 2015 Aug 5;10(8):e0134892. doi: 10.1371/journal.pone.0134892 (PMC4526528; doi:10.1371/journal.pone.0134892)
Supplement: S2 Fig — (PDF) [file pone.0134892.s002.pdf]

Are the SSB-interacting proteins RecO, RecG, PriA and the DnaB-interacting protein Rep bound to progressing replication forks in *Escherichia coli*?

Esma Bentchikou¶, Carine Chagneau¶, Emilie Long¶, Mélody Matelot, Jean-François Allemand and Bénédicte Michel*.

.

Supplementary Figure S2

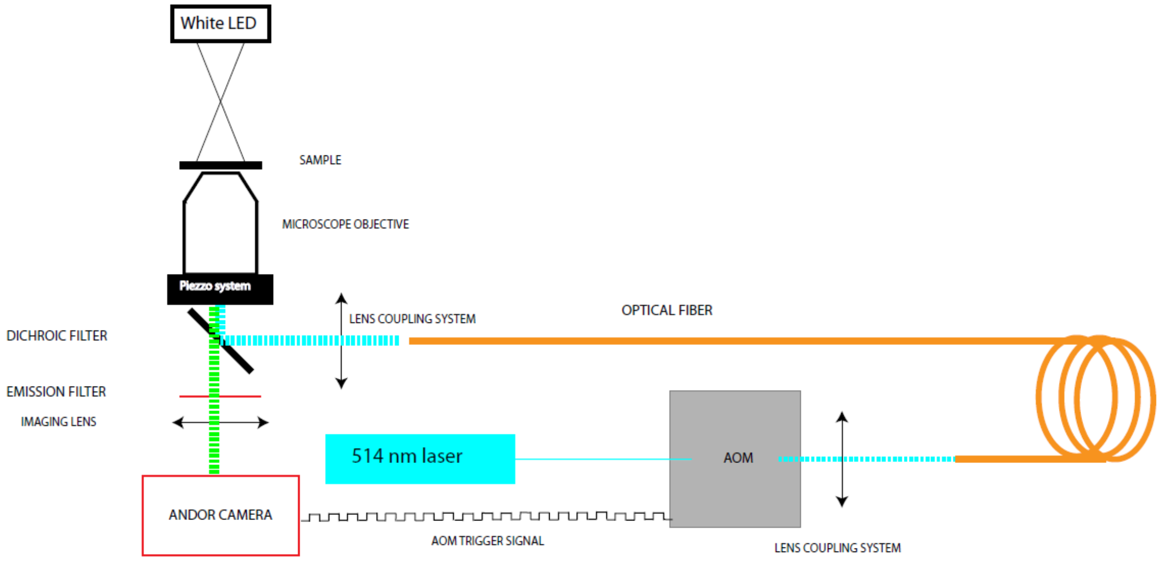

Fig. S2 Optical set-up. The excitation laser beam at 514 nm is modulated at the exit of an Acousto-Optic-Modulator (AOM AOTNFnC-VIS-TN 1001 from AA Opto-Electronics). It is then coupled to the microscope through a 400 µm optical fiber. A dichroic mirror (Di01-R442/514/561 Semrock) directs the beam to the Olympus PlanApo 100X /1.40 objective and the sample. Focus is computer driven with a piezoelectric system (PIFOC polytec PI). Fluorescence light is collected by the objective, filtered thanks to an emission filter (FF01-485/537/627 Semrock), and focused on an EMCCD cooled camera (ANDOR Ultra 897). Camera signal triggers the AOM output and thus illumination is synchronized with detection by the camera. To image bacteria laser illumination is stopped and a white LED (Thorlabs) is used for bright field illumination.
