# Supplementary material for: Are the SSB-Interacting Proteins RecO, RecG, PriA and the DnaB-Interacting Protein Rep Bound to Progressing Replication Forks in Escherichia coli?
Source: PLoS One. 2015 Aug 5;10(8):e0134892. doi: 10.1371/journal.pone.0134892 (PMC4526528; doi:10.1371/journal.pone.0134892)
Supplement: S3 Fig — (PDF) [file pone.0134892.s003.pdf]

Are the SSB-interacting proteins RecO, RecG, PriA and the DnaB-interacting protein Rep bound to progressing replication forks in *Escherichia coli*?

Esma Bentchikou¶, Carine Chagneau¶, Emilie Long¶, Mélody Matelot, Jean-François Allemand and Bénédicte Michel*.

.

Supplementary Figure S3

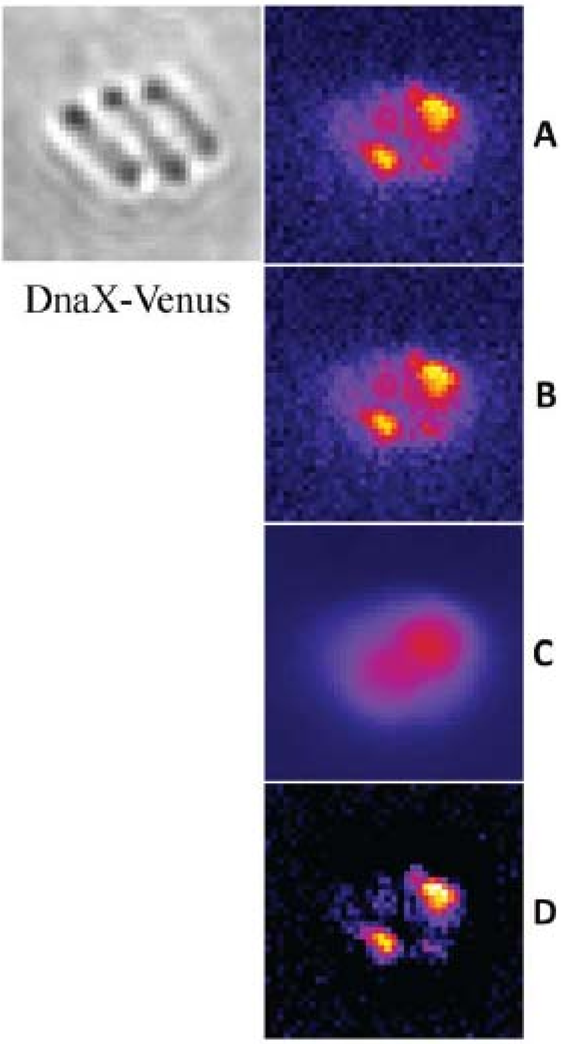

Fig. S3 Image treatment. Left, brightfield image of a group of bacteria expressing DnaX-Venus. Right, the four steps of fluorescent image treatment, shown on the first fluorescent frame. Each treatment is automatically applied to all frames. (A) The original fluorescent image. (B) After treatment with the custom-made Matlab program that only compensate unequal illumination assuming a Gaussian profile illumination. Resulting images were then treated with Image J to remove background. (C) A duplicated image was treated using the ImageJ’s plugin Filter “Sigma Filter Plus”. using the plugin filter parameters: Radius=10 pixels, using pixels within 5 sigma range and Minimum Pixel Fraction = 0.2. (D) The final image with only the fluorescence spots was obtained by subtracting image in C from image in B with ImageJ. Fluorescence spots were defined manually as groups of more than 5 pixels.
