# Supplementary material for: Are the SSB-Interacting Proteins RecO, RecG, PriA and the DnaB-Interacting Protein Rep Bound to Progressing Replication Forks in Escherichia coli?
Source: PLoS One. 2015 Aug 5;10(8):e0134892. doi: 10.1371/journal.pone.0134892 (PMC4526528; doi:10.1371/journal.pone.0134892)
Supplement: S4 Fig — (PDF) [file pone.0134892.s004.pdf]

Are the SSB-interacting proteins RecO, RecG, PriA and the DnaB-interacting protein Rep bound to progressing replication forks in *Escherichia coli*?

Esma Bentchikou[¶], Carine Chagneau[¶], Emilie Long[¶], Mélody Matelot, Jean-François Allemand and Bénédicte Michel*.

.

Supplementary Figure S4

Figure S4. Stable and unstable foci. Using the Image J software, a line is drawn from pole to pole on each cell on the bright field image (left), translated to the foci image (central), and the intensity along the line is measured (graphs on the right). Spots counting less than 5 pixels are not taken into account because such spots could be observed at a relatively high rate in wild-type cells devoid of fluorescent protein and presumably result from autofluorescence. Both the foci image and the intensity graph are used to determine whether a focus is stable (moves by no more than one pixel on at least 3 of the first 4 frames), or unstable (moves by more than one pixel or disappears). The position of the pick on the graph is used to determined whether a focus is central, lateral or neither. (A) Examples of dnaX-Ypet foci. Stable and unstable foci are shown. In these examples both stable foci are central (position 0.4 on the first frame, thus in the 0.4-0.5 window). (B) Examples of $Pr_{rep}$-*venus* and $Pr_{recG}$-*venus* foci. Cells with stable, unstable and with no foci are shown. The stable focus in $Pr_{rep}$-*venus* bacteria 2 is lateral (position 0.15-0.20 on the first frame, thus in the 0.15-0.33 window).

**A**

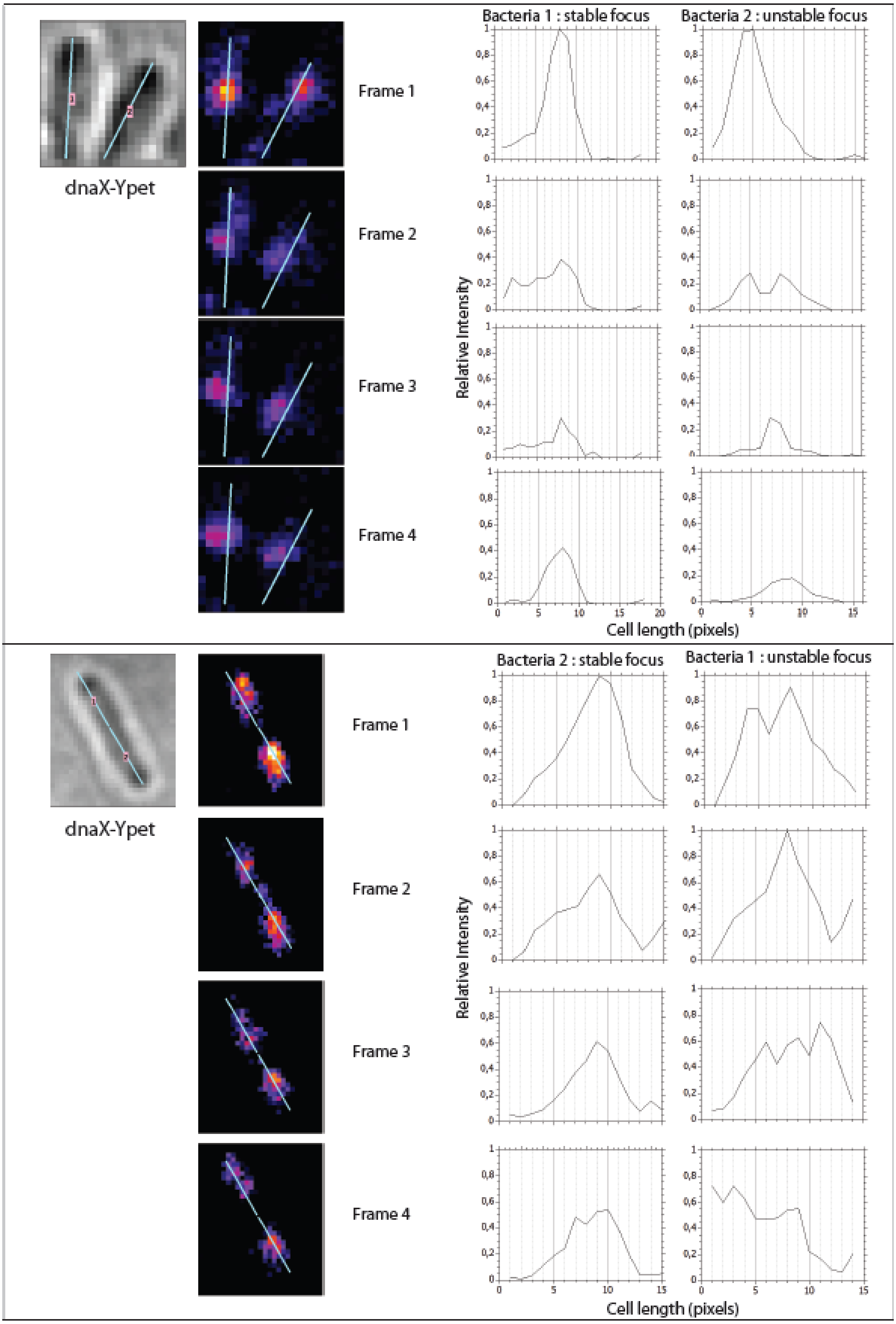
dnaX-Ypet
Frame 1
Frame 2
Frame 3
Frame 4
Bacteria 1 : stable focus
Bacteria 2 : unstable focus
Relative Intensity
Cell length (pixels)
dnaX-Ypet
Frame 1
Frame 2
Frame 3
Frame 4
Bacteria 2 : stable focus
Bacteria 1 : unstable focus
Relative Intensity
Cell length (pixels)

B

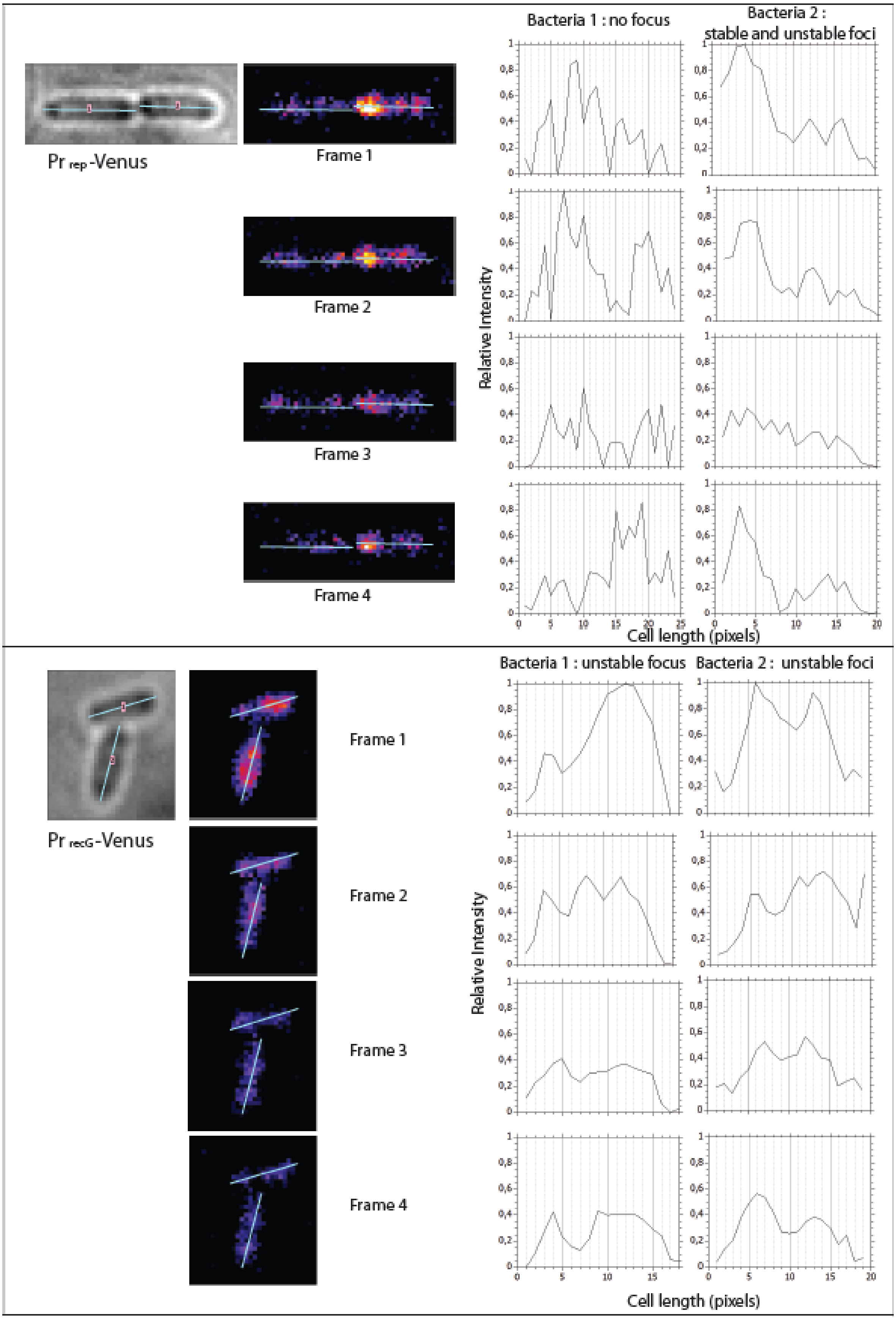
Bacteria 1 : no focus
Bacteria 2 : stable and unstable foci
Pr rep-Venus
Frame 1
Frame 2
Frame 3
Frame 4
Relative Intensity
Cell length (pixels)
Bacteria 1 : unstable focus
Bacteria 2 : unstable foci
Pr recG-Venus
Frame 1
Frame 2
Frame 3
Frame 4
Relative Intensity
Cell length (pixels)
