# Supplementary material for: Are the SSB-Interacting Proteins RecO, RecG, PriA and the DnaB-Interacting Protein Rep Bound to Progressing Replication Forks in Escherichia coli?
Source: PLoS One. 2015 Aug 5;10(8):e0134892. doi: 10.1371/journal.pone.0134892 (PMC4526528; doi:10.1371/journal.pone.0134892)
Supplement: S5 Fig — (PDF) [file pone.0134892.s005.pdf]

Are the SSB-interacting proteins RecO, RecG, PriA and the DnaB-interacting protein Rep bound to progressing replication forks in *Escherichia coli*?

Esma Bentchikou¶, Carine Chagneau¶, Emilie Long¶, Mélody Matelot, Jean-François Allemand and Bénédicte Michel*.

.

Supplementary Figure S5

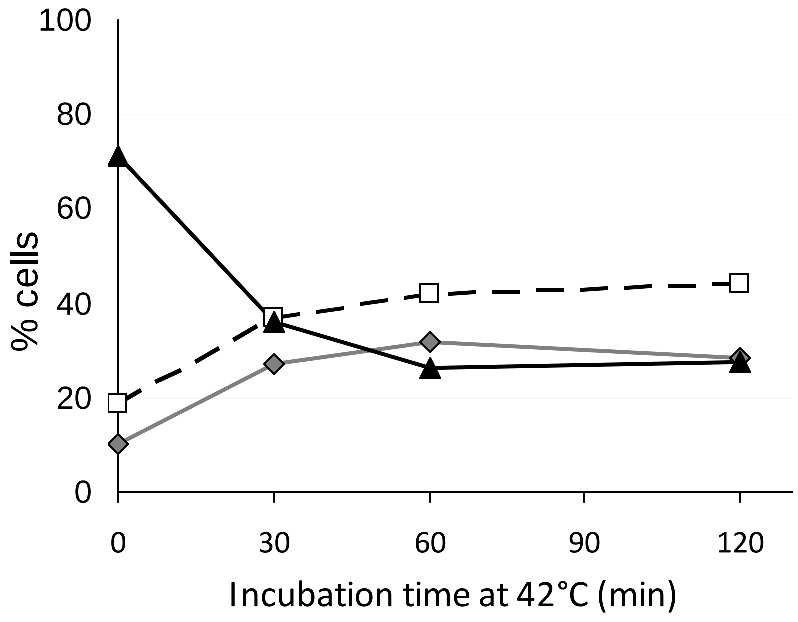

Figure S5. The Y-Pet fluorophore is thermosensitive. Wild-type cells harboring the dnaX-YPet fusion were grown in a microchamber at 30°C and then shifted at time 0 to 42°C. Cells with at least one stable focus (full line, triangles), or only unstable foci (dashed line, squares) and cells with no focus (grey line, diamonds) were counted after 30 min, 60 min and 2 hours of incubation. In this representative experiment, 71% of cells showed at least a stable focus at 30°C. After one hour at 42°C this ratio dropped to 26%, while cells with at least an unstable focus increased from 19% to 42% and cells with no focus increased from 10% to 32%.
